# Unbiased Analysis of Item-Specific Multi-Voxel Activation Patterns Across Learning

**DOI:** 10.3389/fnins.2018.00723

**Published:** 2018-10-04

**Authors:** Hannes Ruge, Eric Legler, Theo A. J. Schäfer, Katharina Zwosta, Uta Wolfensteller, Holger Mohr

**Affiliations:** Department of Psychology, Technische Universität Dresden, Dresden, Germany

**Keywords:** MVPA, pattern similarity, classifier, rapid learning, instruction-based learning, RITL

## Abstract

Recent work has highlighted that multi-voxel pattern analysis (MVPA) can be severely biased when BOLD response estimation involves systematic imbalance in model regressor correlations. This problem occurs in situations where trial types of interest are temporally dependent and the associated BOLD activity overlaps. For example, in learning paradigms early and late learning stage trials are inherently ordered. It has been shown empirically that MVPAs assessing consecutive learning stages can be substantially biased especially when stages are closely spaced. Here, we propose a simple technique that ensures zero bias in item-specific multi-voxel activation patterns for consecutive learning stages with stage being defined by the incremental number of individual item occurrences. For the simpler problem, when MVPA is computed irrespective of learning stage over *all* item occurrences within a trial sequence, our results confirm that a sufficiently large, randomly selected subset of all possible trial sequence permutations ensures convergence to zero bias – but only when different trial sequences are generated for different subjects. However, this does not help to solve the harder problem to obtain bias-free results for learning-related activation patterns regarding consecutive learning stages. Randomization over all item occurrences fails to ensure zero bias when the full trial sequence is retrospectively divided into item occurrences confined to early and late learning stages. To ensure bias-free MVPA of consecutive learning stages, trial-sequence randomization needs to be done separately for each consecutive learning stage.

## Introduction

Multi-voxel pattern analysis (MVPA) has been used to assess whether patterns of BOLD activation across multiple voxels contain content-specific information that cannot be recovered as reliably from individual voxels or from regional mean activations across adjacent voxels (Haxby et al., 2001, 2014; Kriegeskorte et al., 2008; Haynes, 2015). Content-specificity can refer to individual learning items, stimuli, categories, or any other type of discriminable entities.

Recent studies have highlighted the fact that MVPA results can be severely distorted when trial-related BOLD response estimates are based on regression models with systematic, even small, imbalances in the structure of regressor correlations (Mohr et al., 2014; Mumford et al., 2014; Visser et al., 2016). The biggest challenge is faced by experimental procedures in which trial types are inevitably occurring in a fixed order and the corresponding BOLD responses overlap in time (Mumford et al., 2014). For instance, this is the case in learning paradigms where early learning trials necessarily precede later learning trials. Under such conditions it has indeed been shown that MVPA results can be strongly distorted especially when stages are closely spaced (Mohr et al., 2014; Visser et al., 2016). If different trial types of interest can be randomly ordered, distortion due to imbalanced regressor correlations might be less of a concern (Mumford et al., 2014). But even then, regressor correlations might still be residually imbalanced due to imperfect sampling of all possible trial type transitions and even small biases might accumulate substantially over subjects.

**FIGURE 1:**
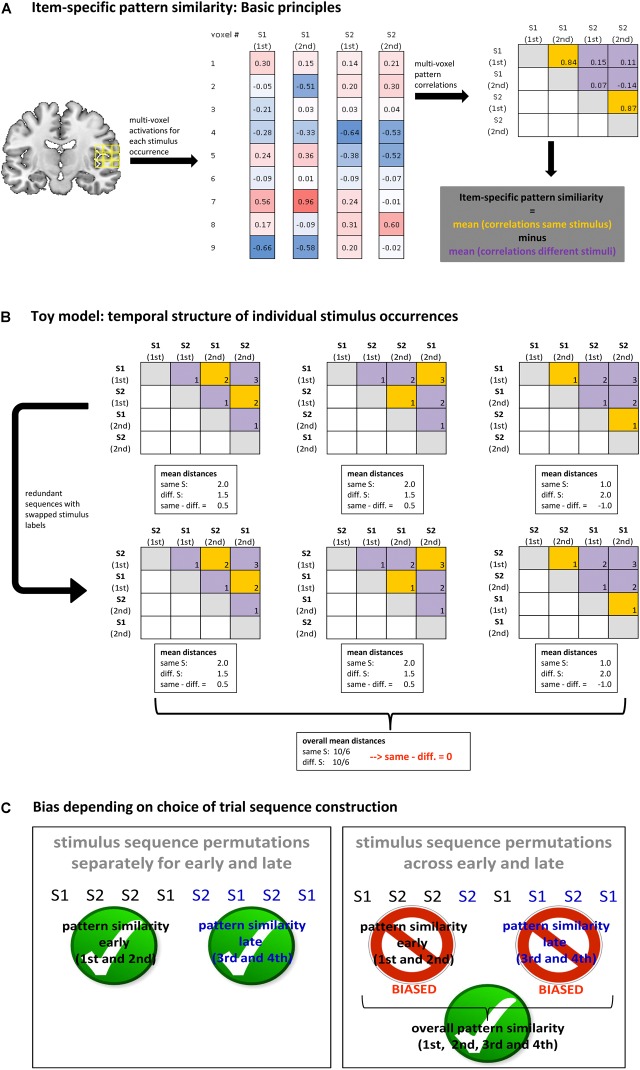
**(A)** Schematic depiction of how item-specific multi-voxel pattern similarity is computed based on the exemplary sequence of two different stimuli each occurring twice. The matrices on the right hand side depict activation pattern correlations for all stimulus combinations. Orange matrix cells denote pattern correlations between same stimuli whereas violet cells denote pattern correlations between different stimuli. Note that sub-diagonal matrix elements are identical with super-diagonal matrix elements and are therefore omitted. Significantly greater mean pattern correlations in orange cells than in violet cells indicate that an activation pattern contains stimulus-specific information. That is, significant pattern similarity is detected. **(B)** Schematic illustration of basic principles based on a toy model involving a set of 2 different stimuli each occurring twice with a constant 1 s stimulus onset asynchrony. Matrices depict absolute stimulus onset differences for all stimulus combinations separately for each possible stimulus sequence permutation. Orange cells denote absolute onset distances between same stimuli whereas violet cells denote absolute onset distances between different stimuli. Stimulus onset differences are used as a proxy (inverse approximation) for regressor correlations. While mean onset differences are different for orange and violet cells for each individual stimulus sequence, on average across all sequence permutations, they are exactly zero (for a formal proof see **Appendix A**). This suggests that on average across sequence permutations, activation pattern correlations are unconfounded with imbalances in onset distances, or regressor correlations for that matter. Hence, no bias in stimulus-specific pattern similarity is expected. **(C)** Schematic illustration highlighting the finding that pattern similarities are biased when subsets of trials are systematically selected (‘partial sequences’) within otherwise well-randomized sequences. For learning paradigms this implicates that stimulus sequences need to be generated independently for consecutive learning stages.

In the present paper, we propose a simple control technique that enables us to determine *unbiased* item-specific multi-voxel activation patterns separately for consecutive learning stages with stage being defined by the incremental number of individual item occurrences. For instance, items occurring the first and second time constitute the early learning stage and items occurring the third and fourth time constitute the late learning stage. Item-specific effects were defined according to the classical rationale (Haxby et al., 2001) that re-occurrences of the same item should produce more similar activation patterns (i.e., voxel vectors correlate more strongly) than occurrences of two different items (see **Figure 1A** for a schematic visualization). Thereby it can be tested if a certain brain region is sensitive to item-specific information. Since item-specific pattern similarities were computed across separate measurement runs in the original Haxby study, distortions are not expected there (cf., Mumford et al., 2014). However, study designs such as the one examined in the present paper are different with regard to two crucial aspects due to the nature of the research question at hand. First, pattern similarities are computed for trial types that are closely spaced and therefore are inevitably intermixed *within* the same measurement run. Second, these different trial types are *sequentially structured*. This is the case for all learning processes involving a high learning rate where learning proceeds across few item repetitions. The assessment of slow learning processes is less critical, as learning stages can easily be segregated into separate measurement runs.

The aim of the present paper is to demonstrate that systematic bias can be avoided even under rapid learning conditions, but only when trial sequences are constructed in a way that respects two prerequisites. To derive these two prerequisites, we distinguish between two problems. The first, *simpler problem* concerns bias-free MVPA for closely spaced trials within runs irrespective of (learning-related) sequential trial structure. The second, *harder problem* concerns bias-free MVPA under consideration of sequential trial structure, that is, when stimulus-specific activation patterns need to be determined separately for consecutive learning stages in order to characterize learning-related changes.

## General Methodology

In a first step, we explore how improper trial sequence construction leads to imbalances in the temporal structure of individual item occurrences. In case of rapid learning designs with strong overlap between BOLD responses, such imbalances are expected to directly translate into imbalances in regressor correlations which have been identified as a root cause of biased MVPA (Mumford et al., 2014). To this end we used a minimalistic toy model which solely considered the lag (i.e., the absolute temporal distance) between (re-)occurrences of same and different stimulus exemplars across a trial sequence.

In a second step, we examined whether the toy model-based predictions regarding proper trials sequence construction would hold under more realistic conditions involving single-trial regression on noisy data. To this end, we performed more elaborate simulations using synthetic fMRI data with realistic fMRI noise added and regression-based single-trial HRF modeling.

In a final third step, we report empirical results to demonstrate the absence of systematic bias in real data when trial sequences are constructed appropriately as suggested by the toy model and the simulations.

Both, the toy model and the simulations were based on different types of trial sequences which were constructed according to the same basic principles. Trial sequences involved different stimuli that could each occur multiple times. To perform item-specific MVPA for a distinct learning stage at least two exemplars per stimulus (i.e., stimulus occurrences) are needed. Hence, the shortest possible learning stage comprised two different stimuli each occurring twice. This minimalistic case was used for illustration purposes in **Figure 1A** to highlight the rationale behind MVPA based on item-specific pattern similarities. Also the toy model started out with this minimalistic case as illustrated in **Figure 1B**. More complex cases could involve more than one learning stage and more than two different stimuli (e.g., two stimuli each occurring four times as depicted in **Figure 1C**).

Both, the toy model and the simulations were used to address both the simpler problem and the harder problem outlined above. Regarding the simpler problem, we assessed the relationship between bias and the temporal structure of *full* trial sequences irrespective of learning stage. Regarding the harder problem, we assessed the relationship between bias and temporal structure of *partial* sequences that resulted from the division of full trial sequences into temporally ordered subsections comprised of early and late learning stage trials, respectively. This latter scenario is of particular interest as it has been shown earlier to be prone to bias when trying to determine differences in activation patterns between successive learning stages (cf., Mohr et al., 2014; Visser et al., 2016). Generally, learning stages were defined by non-overlapping pairs of consecutive stimulus occurrences. The term ‘non-overlapping’ means that the early learning stage was based on stimulus occurrences 1 and 2 whereas the late learning stage was based on stimulus occurrences 3 and 4.

Readers who are not particularly interested in the question how imbalances in the temporal structure of individual item occurrences emerge in the first place, might proceed directly to the simulation section and skip the toy model section.

## Toy Model

### Materials and Methods (Toy Model)

Each toy model variant was simply and solely defined by the set of trial sequences that could be constructed given a certain number of different stimuli and given a certain number of occurrences per stimulus. For simplicity, the stimulus-onset-asynchrony (SOA) was fixed at 1 s for all toy model variants. The trial sequences were then assessed regarding absolute onset distances between (re-)occurrences of same and different stimuli. As illustrated in **Figure 1A** for the case of real experimental (or synthetic) BOLD activation data, stimulus-specific pattern similarities are defined by the mean difference between pattern correlations for re-occurrences of the same stimulus (orange matrix fields) and pattern correlations for occurrences of different stimuli (violet matrix fields). According to Mumford et al. (2014), the mean difference in pattern correlations will be biased if the mean difference between the corresponding regressor correlations is non-zero. Due to BOLD response overlap in case of closely spaced trials, regressor correlation should be a function of trial onset distance. Hence, according to the toy model, unbiased MVPA can be expected if mean onset distances are equal for same and different stimuli.

### Results (Toy Model)

#### Full Sequences Irrespective of Learning Stage (Simpler Problem)

**Figure 1B** (rightmost example) depicts the structure of trial onset distances for the same exemplary stimulus sequence S1-S1-S2-S2 that was used in **Figure 1A** to illustrate the general principles of stimulus-specific pattern similarity. It turns out that mean onset distances are unequal for same stimulus combinations and for different stimulus combinations. Hence, it is expected that stimulus-specific pattern similarity computed for real or synthetic BOLD data are strongly distorted for this exemplary sequence. **Figure 1B** (leftmost and middle examples) also shows that different stimulus sequences imply differently structured onset distances for same and different stimulus combinations, which are also all none-zero. Fortunately, on average across all possible sequences (according to equation 1, exactly 6 unique 4-trial sequences are possible for our toy model) the mean onset difference between same stimulus combinations and different stimulus combinations is exactly zero. Hence, on average across all possible sequences, no distortion of stimulus-specific pattern similarity should be expected – at least according to the simplified toy model. This observation tentatively suggests a first crucial requirement for unbiased MVPA results, namely that all possible stimulus sequences need to be realized equally often. **Appendix A** provides a formal proof that this conclusion holds for trial sequences comprising any number of different stimuli and any number of occurrences per stimulus.

(1) $$\text{Number of unique stimulus sequences}=\frac{{\left(\text{nk}\right)}^{!}}{{\left(\text{k}!\right)}^{n}}$$

with n = number of different stimuli and k = number of occurrences of each stimulus.

**FIGURE 2:**
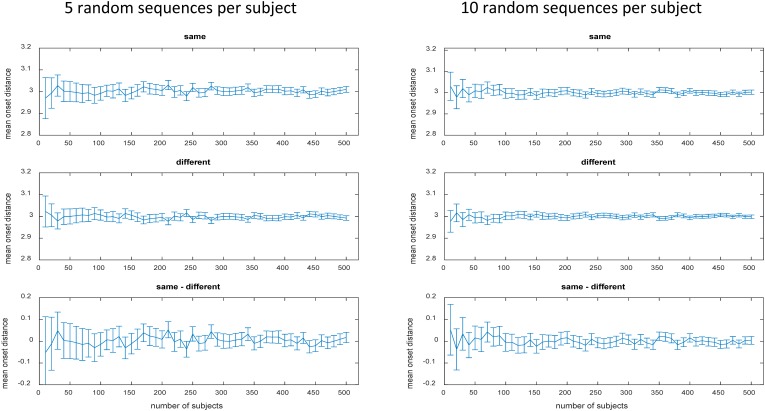
Mean onset distances in seconds for different numbers of randomly selected 8-trial sequences in the toy model (2 stimuli each occurring 4 times). Same, same stimuli; Diff, different stimuli; Error bars represent the 95% confidence interval.

The number of possible sequences is exploding when more than 2 stimuli with more than 2 occurrences are involved (and even more so when the SOA is additionally varied). For instance, the still relatively simple case of 4 stimuli each occurring 3 times already implicates 369,600 unique 12-trial sequences according to equation 1 (see **Table 1** right column). In case of 4 stimuli each occurring 4 times incredible 63,063,000 unique 16-trial sequences are possible. Evaluation of mean onset distances for a few exemplary cases is depicted in **Table 1** to demonstrate that zero bias generalizes to more complex sequences if all unique sequence permutations are realized (for a formal proof see **Appendix A**). However, already in these still relatively simple cases, there are way too many permutations to be all realized in a realistic experiment. Approximation via random sampling from the full set of permutations should be a viable solution if the number of random samples is sufficiently large according to the ‘law of large numbers’ in probability theory. As a tutorial exercise and to give an intuitive impression of zero bias approximation vie random sampling, **Table 1** and **Figure 2** also depict the results of random sampling simulations. **Table 1** shows that zero bias is almost perfectly approximated by random sampling when 5000 subjects are simulated with 30 randomly selected sequences per subject. This relatively large sample size should ensure reasonable power to detect even small biases. **Figure 2** depicts the results for more realistic scenarios of 5 and 10 sequences per subject and sample sizes varying between *N* = 10 and *N* = 500 subjects. Again, zero bias is approximated well, unsurprisingly with smaller error margin for greater *N* and greater number of sequences per subject.

**Table 1:** Mean onset distances in seconds for different types of sequences in the toy model; Random sampling was based on 5000 different sets of 30 randomly selected sequences.

	4-trial sequence (2 stimuli, 2 times)		8-trial sequence (2 stimuli, 4 times)		12-trial sequence (4 stimuli, 3 times)	
	Full permutation	Random sampling	Full permutation	Random sampling	Full permutation	Random sampling
# unique sequences	6	6	70	70	369,600	369,600
Mean distance Same [CI]	1.667 [n.a.]	1.668 [1.666 1.670]	3.000 [n.a.]	3.001 [2.999 3.003]	4.333 [n.a.]	4.334 [4.330 4.337]
Mean distance Diff [CI]	1.667 [n.a.]	1.666 [1.665 1.667]	3.000 [n.a.]	2.999 [2.998 3.001]	4.333 [n.a.]	4.333 [4.333 4.334]
Same-Diff [CI]	0.000 [n.a.]	0.002 [-0.002.006]	0.000 [n.a.]	0.002 [-0.002.005]	0.000 [n.a.]	1.67e-4 [-0.004.004]

*Same, same stimuli; Diff, different stimuli; CI, 95% confidence interval; n.a., not applicable.*

#### Partial Sequences for Consecutive Learning Stages (Harder Problem)

Importantly, the preceding toy model examples have not yet addressed the harder problem, which is, to disentangle without bias multi-voxel activation patterns associated with sequentially structured trial types. This problem can be illustrated using the same type of 8-trial sequences used earlier involving 2 stimuli each occurring 4 times (see **Table 1** middle column). Instead of computing MVPAs across all 4 stimulus occurrences (i.e., neglecting temporal structure), MVPAs can be computed for the same 8-trial sequences by separately assessing and then comparing the early learning stage (stimulus occurrences 1 and 2) and the late learning stage (stimulus occurrences 3 and 4). Clearly, early and late stage trials are sequentially ordered and due to overlapping BOLD responses, it might be difficult to disentangle stage-specific multi-voxel patterns in real or synthetic BOLD data. Indeed, as summarized in **Table 2**, our toy model suggests that MVPA results obtained for each individual learning stage will be biased (same-different onset distances > 0) even though the overall 8-trial sequences were fully permuted (or generated via random sampling).

**Table 2:** Mean onset distances for different types of 8-trial sequences in the toy model.

	8-trial sequence (2 stimuli, 4 times) evaluated occurrences: 1 and 2		8-trial sequence (2 stimuli, 4 times) evaluated occurrences: 3 and 4	
	Full permutation	Random sampling	Full permutation	Random sampling
# unique sequences	70	70	70	70
Mean distance Same [CI]	1.800 [n.a.]	1.799 [1.796 1.802]	1.800 [n.a.]	1.801 [1.799 1.804]
Mean distance Diff [CI]	2.114 [n.a.]	2.115 [2.111 2.118]	2.114 [n.a.]	2.113 [2.110 2.117]
Same-Diff [CI]	-0.314 [n.a.]	-0.316 [-0.321 -0.310]	-0.314 [n.a.]	-0.313 [-0.318 -0.308]

*Same, same stimuli; Diff, different stimuli; CI, 95% confidence interval; n.a., not applicable.*

But how can we explain that MVPA based on 4-trial sequences including two occurrences of 2 stimuli produces zero bias (see **Table 1** left column) whereas MVPA based on the first two occurrences of 2 stimuli as part of longer 8-trial sequences does produce bias (**Table 2**)? A closer inspection reveals that 8-trial sequences suffer from ‘intrusions’ of stimuli occurring the third or fourth time while other stimuli have not yet occurred twice. This causes an imbalance in onset distances compared to 4-trial sequences: specifically, this implies a smaller increase in the mean distance between *same* stimuli (from 1.67 to 1.80 s) than the mean distance between different stimuli (from 1.67 to 2.11 s). In other words, bias will be inevitable if only the first two occurrences of each stimulus are considered while the third and fourth occurrences are omitted during the MVPA.

### Interim Conclusion (Toy Model)

The simplified toy model tentatively suggests two important prerequisite for unbiased MVPA. First, regarding the simpler problem, full permutation of all trial sequences or approximation via random sampling ensures – on average – zero bias even though each individual sequence is associated with bias. Second, regarding the harder problem, the construction of partial sequences by systematic omission of trials occurring in specific sub-sections of otherwise well-randomized full sequences is prohibited when specifying the pattern correlation matrix (or the onset distance matrix, for that matter). For our learning example, this implies that bias can be avoided when 4-trial sequences covering the early learning phase and 4-trial sequences covering the late phase are generated separately and independently and be concatenated afterward. This insight is highlighted schematically in **Figure1C**.

Clearly, the toy model can only deliver tentative conclusions and it remains open whether these hold under more realistic assumptions. For instance, it is unclear whether trial onset distance is really a fully valid proxy for regressor correlation, especially given the non-symmetrical shape of the standard hemodynamic response function (HRF) and given auto-correlated fMRI data. In a second step, we therefore examined the behavior of more realistic simulations involving trial sequences with variable SOAs which were transformed into synthetic BOLD activation data with added real fMRI noise. Furthermore, regular HRF modeling was used to estimate single-trial BOLD responses which were then submitted to MVPA.

## Simulations Based on Synthetic Bold Activation Data

### Materials and Methods (Simulations)

#### General Task

We simulated a rapid learning task in which 4 different stimuli occurred multiple times within a trial sequence. For each pseudo-subject we generated 30 such trial sequences (i.e., 30 unique learning blocks) and simulated a total of 5,000 subjects. This relatively large sample size should ensure reasonable power to detect even small biases under the null-effect simulation. Each of these 150,000 trial sequences was generated independently. The stimulus-onset-asynchrony (SOA) was randomly chosen from an equal distribution of 4 different intervals (2, 3, 4, and 5 s).

#### 8-Trial Sequences With 4 Stimuli Each Occurring 2 Times (Simpler Problem)

In the first family of simulations we examined 8-trial sequences tackling the simpler problem. For each trial, one out of *4* different stimuli was selected randomly from a pre-specified set of *2* occurrences per stimulus. If an item was selected from this set it was not replaced. Since each stimulus occurred just twice, only a single learning stage was defined in this simulation.

#### 16-Trial Sequences With 4 Stimuli Each Occurring 4 Times (Harder Problem)

In the second family of simulations we examined 16-trial sequences tackling the harder problem. For each trial, one out of *4* different stimuli was selected randomly from a pre-specified set of *4* occurrences per stimulus. If an item was selected from this set it was not replaced.

#### Synthetic Data Generation

For each trial we created a synthetic BOLD activation time course which was initially sampled at a ‘microtime’ resolution of TR/16 s as is the default value in the SPM software package. The trial-specific synthetic BOLD responses were obtained by convolving the respective stimulus onset ‘stick’ functions with a canonical event-related BOLD response model using SPM default values. Stimulus-specific multi-voxel activation patterns were generated by creating 4 random number vectors (one for each stimulus) of length 100 (i.e., simulating 100 voxels) which were then multiplied with the respective trial-specific synthetic BOLD responses. To imprint systematic stimulus-specific activation patterns, the same 4 random number vectors were used for each occurrence of the four specific stimuli with Gaussian random noise added to account for the fact that in reality we cannot expect exactly the same pattern to be induced by a re-occurring stimulus. Importantly, we also created synthetic multi-voxel data *without* imprinting systematic stimulus-specific activation patterns. To this end we generated novel random number vectors for each occurrence of the four specific stimuli. In this case, we should not be able detect significant stimulus-specific activation patterns in our simulation – unless the analysis is biased. Hence, in order to detect bias, this null-effect simulation is most crucial.

For each simulated voxel, the sum over all synthetic trial-specific BOLD responses determined the overall BOLD time course for a given trial sequence which was then resampled at *TR* = 2 s.

Finally, to simulate realistic fMRI noise, we used actually measured BOLD data sampled at *TR* = 2 from one of the subjects participating in the study described later in this paper. Instead of using the preprocessed time series data we used unsmoothed residual time series that were created by regressing out task-related activation with a standard high-pass filter of 1/128 Hz using SPM12. For each simulated trial-sequence, fMRI noise time series were newly specified within a randomly chosen time window of the same length as the synthetic time courses from a 100-voxel sphere at a randomly chosen center coordinate.

#### Modeling – Single Trial Estimation

Due to the nature of the task and due to the specific analysis aims, stimulus-specific pattern similarities needed to be based on single-trial estimates. Single-trial activation was modeled using both, the least-squares-all (LSA) approach and the least-squares-single (LSS) approach (Mumford et al., 2012, 2014). The LSA model included regressors for each trial (i.e., 8 regressors for the 8-trial sequences and 16 regressors for the 16-trial sequences), plus a constant. By contrast, an individual LSS model included one regressor for a specific trial and another regressor modeling all other trials, plus a constant. To obtain single-trial estimates for each trial, different LSS models had to be estimated. While LSS modeling is computationally much more time consuming, it has been argued to produce more reliable results (Mumford et al., 2012, 2014). LSA and LSS regressors were created by convolving stick functions with the canonical HRF analogously to the generation of the synthetic BOLD time courses (but without adding noise).

#### Pattern Similarity

Stimulus-specific pattern similarity was computed as depicted in **Figure 1A** based on single-trial estimates for the simulated 100 voxels. Mean correlations for different stimuli (violet in **Figure 1A**) were subtracted from mean correlations for same stimuli (orange in **Figure 1A**). If this difference is significantly larger than zero, a stimulus-specific activation pattern is detected. In case of the null-effect simulation with no systematic stimulus-specific activation pattern being imprinted, a significant effect would imply biased results. Results of one-sample *t*-tests are reported. Note that signed-rank tests yielded qualitatively the same results.

### Results (Simulation)

#### 8-Trial Sequences With 4 Stimuli Each Occurring 2 Times (Simpler Problem)

**FIGURE 3:**
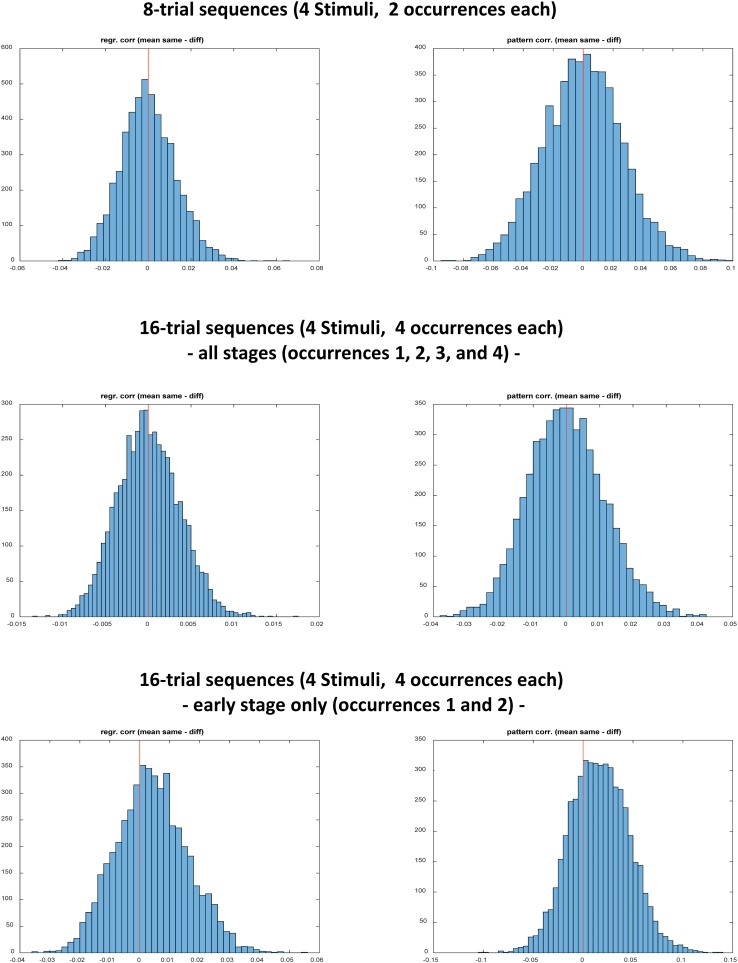
Results of three different simulations all performed without systematic stimulus-specific multi-voxel patterns (null-effect simulation) and based on LSS modeling. Histograms on the left hand side represent the distribution of mean regressor correlation differences between same stimuli and different stimuli. Histograms on the right hand side represent the distribution of mean pattern correlation differences between same stimuli and different stimuli (i.e., stimulus-specific pattern similarities). The red vertical line represents the expected zero mean.

**Table 3** and **Figure 3** summarize the simulation results for 8-trial sequences involving 2 occurrences of each of 4 different stimuli. As predicted by the toy model, there was no significant bias for the null-effect simulation, neither regarding regressor correlations nor regarding pattern similarities. This was true for LSA and LSS single trial modeling. The simulations with imprinted systematic stimulus-specific pattern show that LSA and LSS have comparable power to detect an effect if present.

**Table 3:** Results for different types of simulations all based on 8-trial sequences including 4 stimuli each occurring twice.

8 trial sequences				
**Single-trial modeling**	**Truth: systematic stimulus-specific pattern exists in data**		**Truth: null-effect (i.e., no systematic stimulus-specific pattern in data)**	
	**Regressor correlation**	**Pattern similarity**	**Regressor correlation**	**Pattern similarity**
LSA	*m* = 9e-05	*m* = 0.181	*m* = -8e-05	*m* = -0.0001
	*t* = 0.48; *p* = 0.63	*t* = 533.79; *p* << 0.0001	*t* = -0.44; *p* = 0.66	*t* = -0.41; *p* = 0.68
LSS	*m* = 0.0002	*m* = 0.189	*M* = -5e-05	*M* = 0.0002
	*t* = 0.92; *p* = 0.36	*t* = 499.90; *p* << 0.0001	*t* = -0.27; *p* = 0.79	*t* = 0.42; *p* = 0.67

*m, mean.*

#### 16-Trial Sequences With 4 Stimuli Each Occurring 4 Times (Harder Problem)

**Table 4** and **Figure 3** summarize the simulation results for 16-trial sequences involving 4 occurrences of each of 4 different stimuli. As predicted by the toy model, there was no significant bias for the null-effect simulation if all 4 occurrences were included in the computations (‘overall evaluation’). This was true for both regressor correlations and pattern similarities and for both LSA and LSS single trial modeling. However, also as predicted by the toy model, the simulation results were strongly biased if only the first 2 occurrences were included (‘early stage evaluation’). This was true both for regressor correlations and pattern similarities and for both LSA and LSS single trial modeling. Again, the simulations with imprinted systematic stimulus-specific pattern confirm that LSA and LSS have comparable power to detect an effect if present.

**Table 4:** Results for different types of simulations all based on 16-trial sequences including 4 stimuli each occurring four times.

	16 trial sequences				
	Single-trial modeling	Truth: systematic stimulus-specific pattern exists in data		Truth: null-effect (i.e., no systematic stimulus-specific pattern in data)	
		Regressor correlation	Pattern similarity	Regressor correlation	Pattern similarity
Overall evaluation	LSA	*m* = -2e-05	*m* = 0.056	*m* = -5e-05	*m* = 4e-05
(occurrences 1 to 4)		*t* = -0.35; *p* = 0.73	*t* = 354.90; *p* << 0.0001	*t* = -0.96; *p* = 0.34	*t* = 0.28; *p* = 0.78
	LSS	*m* = 7e-05	*m* = 0.062	*m* = 2e-05	*m* = 4e-05
		*t* = 1.41; *p* = 0.16	*t* = 369.85; *p* << 0.0001	*t* = 0.32; *p* = 0.75	*t* = 0.22; *p* = 0.83
Early stage evaluation	LSA	*m* = 0.004	*m* = 0.065	*m* = 0.004	*m* = 0.007
(occurrences 1 and 2)		*t* = 25.18; *p* << 0.0001	*t* = 163.22; *p* << 0.0001	*t* = 26.15; *p* << 0.0001	*t* = 16.70; *p* << 0.0001
	LSS	*m* = 0.004	*m* = 0.079	*m* = 0.004	*m* = 0.016
		*t* = 25.28; *p* << 0.0001	*t* = 190.50; *p* << 0.0001	*t* = 24.39; *p* << 0.0001	*t* = 38.51; *p* << 0.0001

*m, mean.*

#### The Importance of Proper Trial Sequence Randomization

In the toy model section, we have already highlighted the fact that the number of unique stimulus sequences is increasing non-linearly according to equation 1. For instance, there are already 2520 unique sequences based on 4 stimuli, each occurring twice. This number is increasing dramatically when variable SOAs are additionally considered (e.g., 4 SOA levels implies 6,350,400 unique 8-trial sequences). This was the reason why the simulations performed above were based on approximation via random sampling from the full set of permutations.

As we did for the toy model (see **Table 1** and **Figure 2**), we also evaluated under more realistic conditions, whether this procedure is still a viable option for smaller (and more realistic) sample sizes like *N* = 10 or *N* = 50 instead of *N* = 5000. For each subject, differently sized sets of randomly selected sequences were used (number of sequences per set: 1, 11, 21, 31, and 131, 231, 331, 431, 531, 631, 731, 831, 931). We tested two different scenarios based on the 150,000 8-trial sequences (4 stimuli each occurring 2 twice) generated in the previously completed simulations.

In the first scenario, the same set of random sequences was re-used for each subject. We included this scenario despite common knowledge to instead use different randomization for different subjects in general. The aim was to emphasize the importance of using different randomizations specifically in the context of MVPA (Mumford et al., 2014) where even small biases are known to have big impact compared to conventional univariate analyses. To this end, we randomly selected sets of 8-trial sequences and, importantly, re-used the same set of sequences across all 10 or 50 simulated subjects. In the second scenario, we examined the more reasonable case where different sets of sequences were randomly selected for each subject. To this end, we randomly selected sets of 8-trial sequences with a *novel* set of randomly selected sequences for each of 10 or 50 subjects.

**FIGURE 4:**
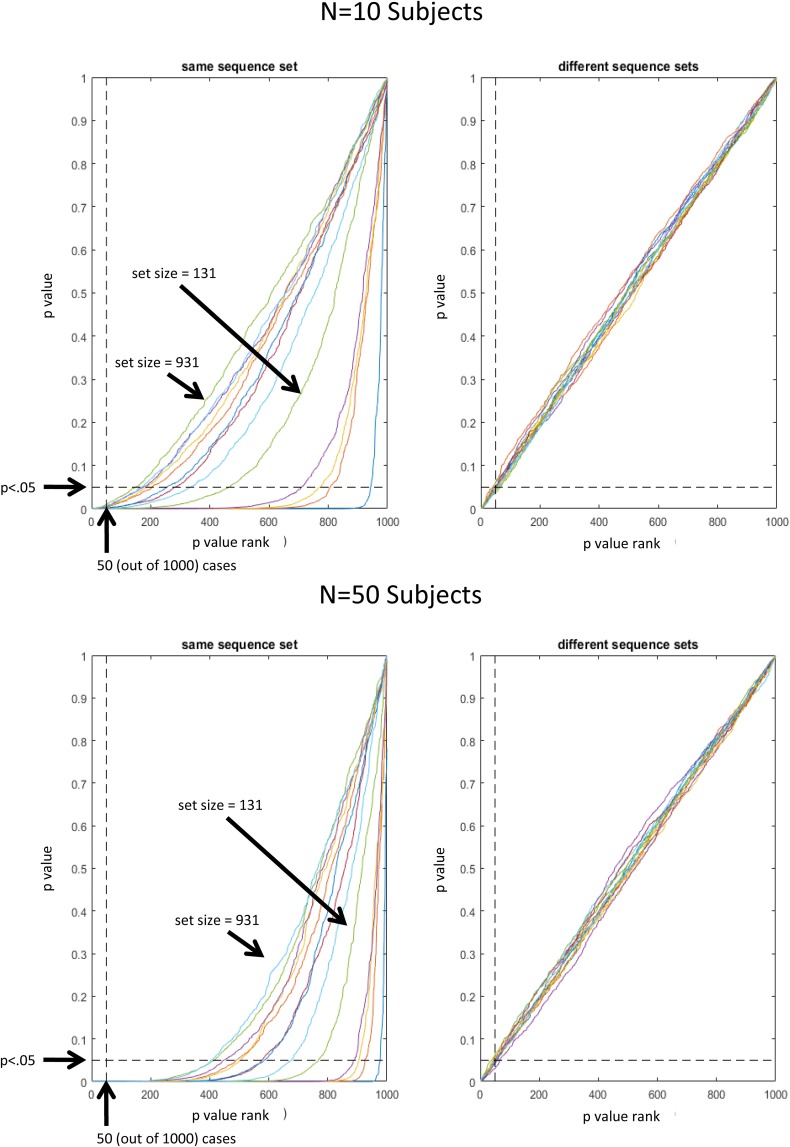
Plot of *p*(t)-values as a function of *p*-value rank. For unbiased results, all *p*-values associated with rank 50 and greater, are expected greater than 0.05. Different lines represent different sequence set sizes (1, 11, 21, 31, and 131, 231, 331, 431, 531, 631, 731, 831, 931).

For both scenarios, for each sequence in a set we took the estimates of stimulus-specific pattern similarities from the previously completed null-effect simulations and added random Gaussian noise (with zero mean and SD equal to the SD across all 150,000 estimated pattern similarities divided by 10). Random noise was generated independently for each subject. Random noise was added to account for the fact that even though a fixed set of trial sequences would translate into a constant potential bias in each subject’s estimated pattern similarities, these pattern similarities would still vary across subjects due to random noise in the subject-specific synthetic data itself. To enable statistical evaluation, the whole procedure was iterated 1000 times (each with a different random selection of sequences per set size). A *t*-test was performed for each iteration and for each set size. The resulting 1000 *p*-values were than assessed regarding above-chance likelihood of *p* < 0.05. Hence, given a distribution of 1000 *p*-values, 50 *p*-values should be < 0.05 by chance. Accordingly, **Figure 4** depicts *p*-values as a function of *p*-value rank. That is, for unbiased results, all *p*-values associated with rank 50 and greater, should be greater than 0.05. This representation of results clearly shows that significant bias was present for all set sizes when a fixed set of randomly selected sequences was re-used for all 10 or all 50 subjects (left column). In contrast, when unique sets of randomly selected sequences were used for each subject, bias was not detectable (right column). Thus, it seems safe to conclude that stimulus-specific activation patterns can be assessed without bias, if sequences are generated randomly within and, importantly, also across subjects. Otherwise, the sensitivity of MVPA for even small biases implies a great danger for bias accumulation when the same randomization is re-used for all subjects (accordingly, the bias becomes even larger for larger *N*).

## Real Data Example

### Materials and Methods (Real Data)

#### Participants

The sample consisted of 24 participants (12 female, 12 male; mean age: 24.4 years, range 20–33 years). All participants were right-handed, neurologically healthy had normal or corrected vision including normal color vision. The experimental protocol was approved by the Ethics Committee of the Technische Universität Dresden and conformed to the World Medical Association’s Declaration of Helsinki. All participants gave written informed consent before taking part in the experiment and were paid 8 Euros per h for their participation or received course credit.

#### Task

The experimental task had roughly the same general structure as the simulated task. Each subject was asked to work through 36 learning blocks each composed of an initial instruction phase and a subsequent 16-trial implementation phase. MVPA was exclusively applied to the implementation phase. Each block involved a unique set of 4 different 2-syllable German nouns. During the implementation phase each noun was presented 4 times and required a button press response consistently with one out of three right hand fingers. Prior to the start of a novel trial sequence, subjects completed the instruction phase in which they were informed about the correct responses to be executed later on during the implementation phase. The start of the impending instruction phase was announced by the German word for “Memorize” (“Einprägen”) displayed in red for 2 s, immediately followed by the presentation of the first instructed noun. Each 16-trial implementation phase was announced by the German word for ‘Implement’ (‘Ausführen’) displayed in green for 2 s.

As suggested by the simulation results, each 16-trial sequence was composed of two independently generated 8-trial sequences, each comprising two occurrences of each of the four nouns. Thereby we wanted to ensure that pattern similarities could be estimated without bias separately for the early learning phase (occurrences 1 and 2) and for the late learning phase (occurrences 3 and 4). There was no performance feedback after individual trials. The SOA varied randomly between 2 and 4 s in 0.5 s steps. The SOA interval was inserted *before* the start of a new trial to ensure that there was also random jitter between the end of the instruction phase and the beginning of the first implementation trial. After a variable delay of 2 or 4 s relative to the end of the last trial, the Implementation phase ended with a display of the mean performance accuracy computed across the preceding 16 trials.

The start of the instruction phase announcement was delayed by a variable delay of 2 or 4 s relative to the start of a new measurement run or relative to the end of the preceding implementation phase. During instruction, the 4 nouns were presented in rapid succession framed by two vertical lines to the left and to the right of the word. If a noun was closer to the left vertical bar, this indicated an index finger response. If a noun was closer to the right vertical bar, this indicated a ring finger response. If a noun was equally close to both vertical bars, this indicated a middle finger response. During instruction, response execution was not allowed yet. We only recruited right-handed subjects who were asked to use the right hand fingers for responding. Note that the instruction phase was not part of the MVPA.

There were two difficulty conditions which only differed in the number of instructed nouns, but not in the number of actually implemented nouns. In the difficult condition, 10 nouns were instructed and each word was display for 1 s. In the easy condition, 4 nouns were instructed and each word was displayed for 2 s. In either case, 4 nouns were presented during the 16-trial implementation phase. There was an equal number of 18 blocks per difficulty condition. For the purpose of the present paper, easy and difficult blocks were lumped together.

#### Data Acquisition

The 36 learning blocks were realized in three consecutive measurement runs each comprising 12 learning blocks. Each run lasted approximately 13 min. Easy and difficult learning blocks were pseudo-randomized such that the same number of 6 blocks per condition was realized. Also, the random delay before the start of each novel instruction phase and the delay before performance feedback was pseudo-randomized such that each SOA level occurred equally often (3 times) for each difficulty condition. MRI data were acquired on a Siemens 3T whole body Trio System (Erlangen, Germany) with a 32 channel head coil. Ear plugs dampened scanner noise. After the experimental session structural images were acquired using a T1-weighted sequence (*TR* = 1900 ms, *TE* = 2.26 ms, *TI* = 900 ms, flip = 9°) with a resolution of 1 mm × 1 mm × 1 mm. Functional images were acquired using a gradient echo planar sequence (*TR* = 2000 ms, *TE* = 30 ms, flip angle = 80°). Each volume contained 32 slices that were measured in ascending order. The voxel size was 4 mm × 4mm × 4 mm (gap: 20%). In addition, field maps were acquired with the same spatial resolution as the functional images in order to correct for inhomogeneity in the static magnetic field (*TR* = 352 ms, short *TE* = 5.32 ms, long *TE* = 7.78 ms, flip angle = 40°). The experiment was controlled by E-Prime 2.0.

#### Preprocessing

The fMRI data were analyzed using SPM12 running on MATLAB R2016a. First, the functional images were slice time corrected, spatially realigned and unwarped using the acquired field maps. Each participant’s structural image was co-registered to the mean functional image and segmented. Spatial normalization to MNI space was performed by applying the deformation fields generated by the segmentation process to the functional images (resolution: 3 mm × 3 mm × 3 mm). Images were not additionally smoothed.

#### Modeling

Standard General Linear Model (GLM) analysis was performed within the SPM12 framework including the standard 1/128 Hz high-pass filter. BOLD activations during the 16-trial implementation phase were modeled analogously to the simulations by using single trial regressors for each of the 3 measurement runs obtained by convolution with the SPM-default HRF (16^∗^12 = 184 single trial regressors per run). Since the simulations did not show any systematic differences between LSS and LSA modeling we used the LSS model which has previously been reported to be more powerful (Mumford et al., 2012, 2014). In addition, we included regressors for the instruction phase and for the performance feedback at the end of each implementation phase. To appropriately capture BOLD activation during the instruction phase, spanning either 12 s (easy condition) or 14 s (difficult condition), we used Fourier basis set regressors including 20 different sine-wave regressors spanning 44 s which were time-locked to the onset of the start of the instruction phase. Using a Fourier basis set has the advantage to flexibly model any BOLD response shape associated with the extended instruction phase without making prior shape assumptions. Advantage over FIR modeling is that a Fourier basis set can easily operate at micro-time resolution (SPM default TR/16) whereas FIR cannot (Henson and Friston, 2007). Performance feedback was modeled with a standard event-related HRF function time-locked to the onset of the feedback screen.

#### MVPA

Multi-voxel pattern analysis was based on single-trial beta estimates analogously to the simulations. As described in the simulations section, stimulus-specific multi-voxel activation patterns were determined by subtracting mean pattern correlations between beta estimates for different learning items from mean pattern correlations between beta estimates of re-occurrences of same learning item. As in the simulations, we did this separately for the early learning stage (occurrences 1 and 2) and the late learning stage (occurrences 3 and 4). These pattern similarity values were projected to each voxel by use of the searchlight approach (Kriegeskorte et al., 2006) which was implemented using the CosmoMVPA toolbox (Oosterhof et al., 2016). Spherical searchlights with 3 voxel radius were used.

Two types of MVPA were performed. A first MVPA was geared toward detecting bias if present. We therefore assessed pattern similarities in white matter were no significant effect was expected in case of bias-free MVPA. To this end, we computed voxel-wise searchlight MVPAs for each subjects’ individually segmented white matter volumes. The mean over all voxel-wise pattern similarity values within white matter was then computed for each subject and assessed via two-tailed one sample *t*-tests. A second MVPA aimed at demonstrating significant pattern similarity effects in meaningful brain regions. For instance, as different nouns were consistently associated with different right hand finger response, significant pattern similarity effects should be expected in the left motor cortex but not in the right motor cortex.

### Results (Real Data)

**FIGURE 5:**
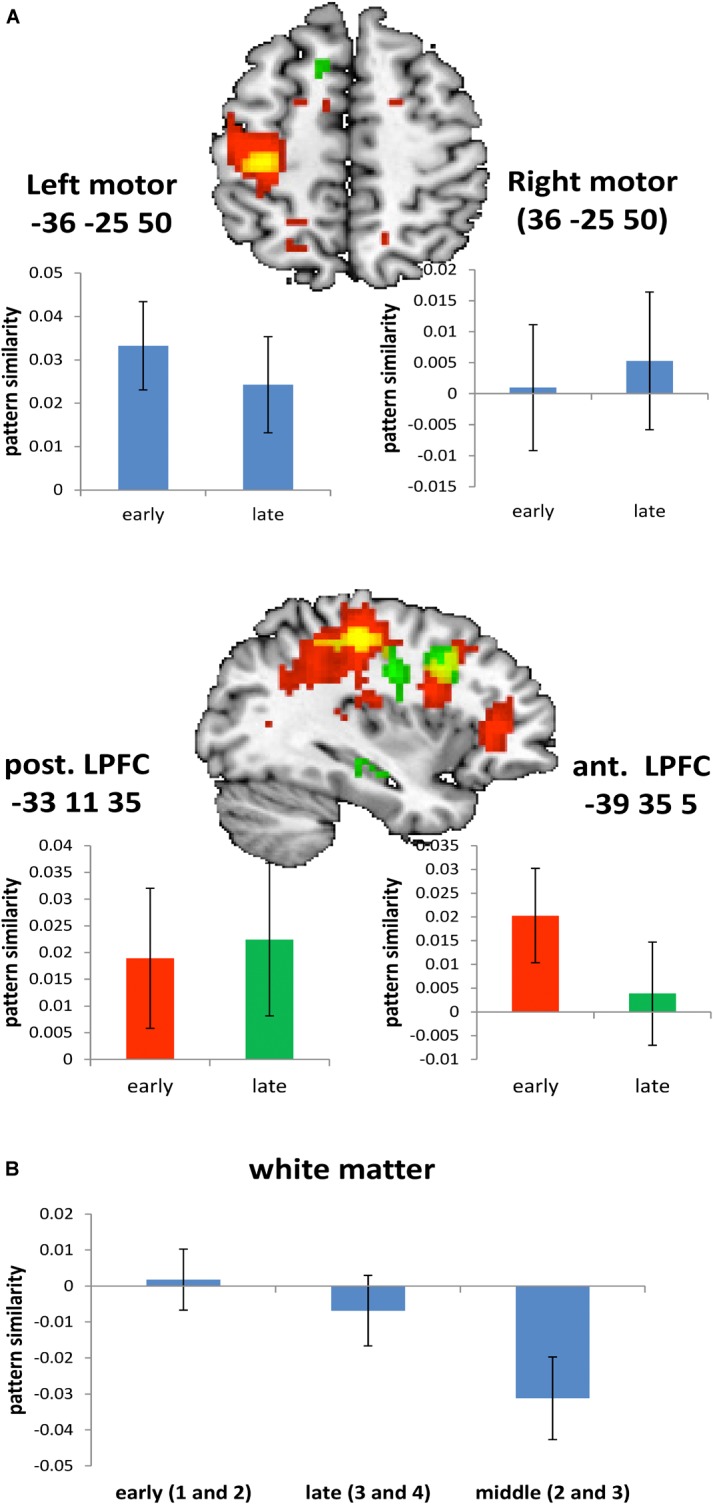
Pattern similarity results based on real data; error bars represent 90% confidence interval. **(A)** T-maps representing early stage (red) and late stage (green) pattern similarities and their overlap (yellow), each thresholded at *t* > 2.0 for visualization. **(B)** Searchlight pattern similarity results averaged across all white matter voxels.

Regarding white-matter MVPA, *t*-tests for both the early learning stage (occurrences 1 and 2) and the late learning stage (occurrences 3 and 4) were non-significant (**Table 5** and **Figure 5**). This suggests the absence of significant bias at least of a size that could have been detected with given statistical power. By contrast, when MVPA was based on occurrences 2 and 3 results turned out to be strongly and significantly biased. This was expected based on the simulations as trial sequences were properly randomized only for within the early and within late learning stages.

**Table 5:** Pattern similarity results based on real data.

	Early (1 and 2)			Late (3 and 4)			Middle (2 and 3)		
	Pattern similarity	*t*	*p*(t)	Pattern similarity	*t*	*p*(t)	Pattern similarity	*t*	*p*(t)
White matter [Mean over all voxels]	0.002	0.44	0.67	0.001	0.26	0.80	- 0.0310	- 5.61	1.04e-05
Left motor cortex [MNI: -36 -25 50]	0.033	5.37	9.36e-06	0.024	3.58	7.93e-04	- 0.01	- 1.25	0.12
Right motor cortex [MNI: 36 -25 50]	0.001	0.12	0.45	0.005	0.78	0.23	- 0.035	- 3.39	0.001
Left post. LPFC [MNI: -33 11 35]	0.019	2.09	0.024	0.022	3.05	0.003	- 0.036	- 3.59	0.001
Left ant. LPFC [MNI: -39 35 5]	0.020	4.63	5.88e-05	0.004	0.48	0.32	- 0.035	- 3.66	0.001

The whole-brain searchlight analysis of pattern similarities revealed several significant clusters in meaningful regions. Two regions were identified based on a one-tailed global conjunction *t*-test across early and late learning stage similarity estimates. This included the left motor cortex (MNI -39 35 5; *t*_2,46_ = 3.14; *p*(t) < 2.18e-06) and the left posterior lateral PFC (MNI -33 11 35; *t*_2,46_ = 2.38; *p*(t) < 1.17e-04). For comparison, **Table 5** and **Figure 5** also include the null-results for the homolog right motor cortex. Note that statistics reported in **Table 5** refer to standard one-tailed *t*-tests applied to pattern similarities regarding each learning stage considered alone (i.e., not in conjunction). Additionally, the left anterior lateral PFC was identified based on a one-tailed *t*-test specifically for the early learning stage (MNI -39 35 5; *t*_1,23_ = 4.63; *p*(t) < 5.88e-05).

In summary, these finding suggest that MVPA based on properly generated random trial sequences ensures protection from bias-induced false positives and at the same time enables us to detect true effects if present.

## Discussion

Previous work has highlighted that MVPA can be severely biased due to even small imbalances in regressor correlations (Mohr et al., 2014; Mumford et al., 2014; Visser et al., 2016). This contrast with conventional univariate analyses where regressor correlation is known to be less of a concern, even for extreme cases with fixed temporal order of closely spaced within-trial events (Ollinger et al., 2001; Serences, 2004; Ruge et al., 2009). This benign behavior in the univariate case is due to the fact that regressor correlation increases beta estimate variance but preserves the mean (Mumford et al., 2015).

The potentially devastating MVPA-specific problem can be easily avoided when the experimental paradigm allows for a complete separation of BOLD activity induced by the contrasted trial types either across runs (e. g., Haxby et al., 2001; Li et al., 2009) or across-subjects (Mohr et al., 2015). The results presented in the present paper demonstrate that MVPA bias can also be avoided in situations where the contrasted trial types are temporally dependent and even when BOLD responses are overlapping. Importantly, this only holds if two pre-requisites are met.

First, activation patterns can be assessed without bias if trial sequences are generated randomly within and, importantly, also across subjects. This confirms earlier conclusions by Mumford et al. (2014) and highlights the sensitivity of MVPA for even small biases accumulating over subjects when the same randomization is re-used for all subjects (note that the problem grows with increasing *N*).

Second, substantial MVPA bias can occur when *partial sequences* are constructed based on specific subsets of trials which are systematically selected within otherwise well-randomized *full sequences*. In the learning example presented here, this implies that zero bias for consecutive learning stages can be ensured by generating separate random trial sequences for each stage. Importantly, this is true even in the presence of overlap between early and late stage BOLD activity which is inevitable for rapid learning processes where learning stages occur within the same measurement run. While certain task constraints might intrinsically imply obedience to this principle (e.g., Kahnt et al., 2011) other studies might have run into trouble due to sub-optimal trial sequence construction. The present results urge for an explicit consideration of proper trial sequence construction.

Clearly item-specific MVPA depends on reliable estimates of single-trial BOLD activation. Our simulations implemented different variants of single-trial GLMs using either the LSS or the LSA methods proposed earlier (Mumford et al., 2012; Turner et al., 2012). These two approaches did reveal quite similar results in the present case. Nevertheless, recent papers have suggested advantages and disadvantages of either approach depending on a number of additional design parameters (Abdulrahman and Henson, 2016; Zeithamova et al., 2017). It might be a worthwhile aim for future research to examine the implications of certain design parameter choices and certain regularization procedures (Mumford et al., 2012) on single trial estimation efficiency and its ramifications on learning-related MVPA.

## Conclusion

In conclusion, the feasibility of MVPA in the context of temporally dependent trial types can be viewed more optimistically than previously thought (Mohr et al., 2014; Visser et al., 2016). In fact, our example demonstrates that the existence of sequential dependencies does not *per se* exclude bias-free MVPA even when trials are not separated across different runs. Hence, MVPA can be employed for the investigation of rapid learning processes and it is not restricted to paradigms where slowly evolving learning stages naturally occur in well-separated runs (e.g., Li et al., 2009). Moreover, it becomes feasible to compute *learning stage-specific* correlations between pattern similarity measures and subsequent behavioral measures of learning success as an extension of previous successful, yet stage-unspecific, applications of this general approach (e.g., Xue et al., 2010; Atir-Sharon et al., 2015). Clearly, within the broader context of learning, the present conclusions might not always be directly applicable. In particular, this holds for learning processes that cannot easily be defined by the number of stimulus occurrences. For instance, if learning stage is defined by some behaviorally derived parameter, this cannot be considered already during the construction of trial sequences prior to the start of the experiment. Whether MVPA bias could be controlled via adaptive on-line sequence construction would require in depth analysis of the specific experimental setting.

## Author Contributions

HR, HM, and EL developed and tested the analysis methods. HR, TS, and UW designed conducted, and analyzed the empirical study. HR, HM, EL, TS, KZ, and UW drafted the work and revised it critically for important intellectual content.

## Conflict of Interest Statement

The authors declare that the research was conducted in the absence of any commercial or financial relationships that could be construed as a potential conflict of interest.
