# Supplementary material for: Unbiased Analysis of Item-Specific Multi-Voxel Activation Patterns Across Learning
Source: Front Neurosci. 2018 Oct 4;12:723. doi: 10.3389/fnins.2018.00723 (PMC6180163; doi:10.3389/fnins.2018.00723)
Supplement: Supplementary file 1 [file Data_Sheet_1.PDF]

Appendix: Proof of balanced distances between same and different stimuli

Let n be the number of different stimuli, and k be the number of appearances of each stimulus (with $n \geq 2$ and $k \geq 2$). Furthermore, let

$$T = \{1, 2, 3, \ldots, nk\}$$

be the set of all trial numbers,

$$S = (S_1, S_2, S_3, \ldots, S_{nk})$$

a sequence of stimuli, and

$$M = \{(S_1, S_2, S_3, \ldots, S_{nk}) \,|\, \text{Each of the } n \text{ different stimuli appears exactly } k \text{ times}\}$$

the set of all stimulus sequences of interest.

Then it is

$$\#M = \binom{nk}{k}\binom{(n-1)k}{k}\binom{(n-2)k}{k}\cdots\binom{k}{k} = \frac{(nk)!}{(k!)^n}$$

Indeed, generating such sequences is equivalent to drawing from T without replacement, with the first k drawings indicating the trial numbers for stimulus 1, the second k drawings the trial numbers for stimulus 2 and so on up to stimulus n.

Furthermore, let

$$A = \{m \in \mathcal{P}(T) \,|\, \#m = 2\} = \big\{\{1,2\}, \{1,3\}, \ldots, \{1, nk\}, \{2,3\}, \{2,4\}, \ldots, \ldots, \{nk-1, nk\}\big\}$$

be the set of trial pairs. It is $\#A = \binom{nk}{2}$.

For a given stimulus sequence $S = (S_1, S_2, S_3, \ldots, S_{nk})$, we define

$$I_S = \{\{i, j\} \in A \,|\, S_i = S_j\}$$

as the set of all trial pairs with identical stimuli, and

$$D_S = \{\{i, j\} \in A \,|\, S_i \neq S_j\}$$

as the set of all trial pairs with different stimuli.

Generally, it is $I_S \,\dot\cup\, D_S = A$, and for any $S \in M$, it is $\#I_S = n\binom{k}{2}$ and $\#D_S = k^2\binom{n}{2}$.

We want to show that across all sequences $S \in M$, the average distance between identical stimuli is equal to the average distance between different stimuli, i.e. we want to show that

$$\sum_{S\in M}\left(\frac{1}{\#I_S}\sum_{\{i,j\}\in I_S} d(S_i,S_j)\right) = \sum_{S\in M}\left(\frac{1}{\#D_S}\sum_{\{i,j\}\in D_S} d(S_i,S_j)\right)$$

with the distance being defined as $d(S_i,S_j) = |i-j|$.

It is

$$\sum_{S\in M}\left(\frac{1}{\#I_S}\sum_{\{i,j\}\in I_S} d(S_i,S_j)\right) = \sum_{S\in M}\left(\frac{1}{\#D_S}\sum_{\{i,j\}\in D_S} d(S_i,S_j)\right)$$

$$\Leftrightarrow \quad \sum_{S\in M}\sum_{\{i,j\}\in I_S} d(S_i,S_j) = \frac{\#I_S}{\#D_S}\sum_{S\in M}\sum_{\{i,j\}\in D_S} d(S_i,S_j)$$

$$\Leftrightarrow \quad \sum_{S\in M}\sum_{\{i,j\}\in I_S} d(S_i,S_j) = \frac{\#I_S}{\#D_S}\left(\sum_{S\in M}\sum_{\{i,j\}\in A} d(S_i,S_j) - \sum_{S\in M}\sum_{\{i,j\}\in I_S} d(S_i,S_j)\right)$$

$$\Leftrightarrow \quad \sum_{S\in M}\sum_{\{i,j\}\in I_S} d(S_i,S_j) = \frac{\#I_S}{\#A}\sum_{S\in M}\sum_{\{i,j\}\in A} d(S_i,S_j)$$

$$\Leftrightarrow \quad \sum_{\{i,j\}\in A}\sum_{\substack{S\in M,\\ S_i=S_j}} d(S_i,S_j) = \sum_{\{i,j\}\in A}\frac{\#I_S}{\#A}\sum_{S\in M} d(S_i,S_j)$$

Hence, it is sufficient to show that

$$\forall \{i,j\}\in A: \quad \sum_{\substack{S\in M,\\ S_i=S_j}} d(S_i,S_j) = \frac{\#I_S}{\#A}\sum_{S\in M} d(S_i,S_j)$$

For any $\{i, j\} \in A$, it is

$$\sum_{\substack{S \in M, \\ S_i = S_j}} d(S_i, S_j) = \frac{\#I_S}{\#A} \sum_{S \in M} d(S_i, S_j)$$

$$\Leftrightarrow \qquad d(S_i, S_j) \sum_{\substack{S \in M, \\ S_i = S_j}} 1 = d(S_i, S_j) \frac{\#I_S}{\#A} \sum_{S \in M} 1$$

$$\Leftrightarrow \qquad \#\{S \in M \,|\, S_i = S_j\} = \frac{\#I_S}{\#A} \#M$$

It is

$$\#\{S \in M \,|\, S_i = S_j\} = n \binom{nk-2}{k-2} \binom{(n-1)k}{k} \binom{(n-2)k}{k} \cdots \binom{k}{k}$$

and

$$\frac{\#I_S}{\#A} \#M = \frac{n\binom{k}{2}}{\binom{nk}{2}} \binom{nk}{k} \binom{(n-1)k}{k} \binom{(n-2)k}{k} \cdots \binom{k}{k}$$

The first identity can be seen by assigning some stimulus, for instance stimulus 1, to trial numbers i and j. Then the remaining trial numbers for stimulus 1 have to be drawn from $T \setminus \{i, j\}$, i.e. there are $\binom{nk-2}{k-2}$ ways to distribute the remaining instances of stimulus 1. Subsequently, the k appearances for each of the remaining $n-1$ stimuli have to be drawn successively from the respective set of remaining trial numbers. Moreover, each of the n different stimuli has to be assigned to the trial numbers $\{i, j\}$, resulting in the factor n.

Hence, it remains to show that

$$n \binom{nk-2}{k-2} = \frac{n\binom{k}{2}}{\binom{nk}{2}} \binom{nk}{k}$$

which can be seen by expanding both sides:

$$\frac{n(nk-2)(nk-2-1)(nk-2-2)\ldots(nk-2-(k-2)+1)}{(k-2)!}$$

$$= \quad \frac{n\frac{k(k-1)}{2}}{\frac{nk(nk-1)}{2}} \frac{nk(nk-1)(nk-2)\ldots(nk-k+1)}{k!}$$
